# The efficacy of early office hysteroscopy in preventing intrauterine adhesions after abortion: a randomized controlled trial

**DOI:** 10.1186/s12905-024-03247-0

**Published:** 2024-07-13

**Authors:** Ni-Chin Tsai, Yu-Yang Hsiao, Yu-Ting Su, Yu-Ju Lin, Fu-Tsai Kung, Ping-Ho Chen, Kuo-Chung Lan

**Affiliations:** 1 Department of Obstetrics and Gynecology, Kaohsiung Chang Gung Memorial Hospital, Chang Gung University College of Medicine, Kaohsiung 123 Ta-Pei Road, Niao-Sung District, Kaohsiung City, Taiwan; 2 Graduate Institute of Clinical Medicine, College of Medicine, Kaohsiung Medical University, 100, Shih-Chuan 1st Road, Kaohsiung, 80708 Taiwan; 3 Center for Menopause and Reproductive Medicine Research, Kaohsiung Chang Gung Memorial Hospital, Kaohsiung, 83301 Taiwan; 4 Department of Obstetrics and Gynecology, Jen-Ai Hospital, Taichung, 41257 Taiwan

**Keywords:** Fertility outcome, Hysteroscopy, Induced abortion, Intrauterine adhesions, Suction dilatation and curettage

## Abstract

**Background:**

Intrauterine adhesions (IUA) are a challenging clinical problem in reproductive infertility. The most common causes are intrauterine surgery and abortions. We aimed to investigate whether early second-look office hysteroscopy can prevent IUA.

**Methods:**

A single-center, prospective, two-armed, randomized controlled trial was designed to explore the efficacy of early office hysteroscopy after first-trimester induced abortion (suction dilatation and curettage [D&C]) and to further analyze fertility outcomes. Women aged 20–45 years undergoing suction D&C and desiring to conceive were recruited. Between October 2019 and September 2022, 66 women were enrolled, of whom 33 were allocated to group A (early hysteroscopy intervention). The women in intervention group A were planned to receive 2 times of hysteroscopies (early and late). In group B, women only underwent late (6 months post suction D&C) hysteroscopy.

**Results:**

The primary outcome was the IUA rate assessed using office hysteroscopy 6 months after artificial abortion. Secondary outcomes included menstrual amount/durations and fertility outcomes. In intervention group A, 31 women underwent the first hysteroscopy examination, and 15 completed the second. In group B (late hysteroscopy intervention, 33 patients), 16 completed the hysteroscopic exam 6 months after an artificial abortion. Twenty-one women did not receive late hysteroscopy due to pregnancy. The IUA rate was 16.1% (5/31) at the first hysteroscopy in group A, and no IUA was detected during late hysteroscopy. Neither group showed statistically significant differences in the follow-up pregnancy and live birth rates.

**Conclusions:**

Early hysteroscopy following suction D&C can detect intrauterine lesions. IUA detected early by hysteroscopy can disappear on late examination and become insignificant for future pregnancies. Notably, the pregnancy outcomes showed a favorable trend in the early hysteroscopy group, but there were no statistically significant differences.

**Trial registration:**

ClinicalTrials.gov, ID: NCT04166500. Registered on 2019-11-10. https://clinicaltrials.gov/ct2/show/NCT04166500.

## Background

Intrauterine adhesions (IUA) are a challenging clinical problem in reproductive infertility [1]. They are typically caused by trauma to the endometrial basal layer during curettage, endometritis, and hysteroscopic myomectomy [2, 3]. Therefore, hysteroscopy is presently the main treatment for IUA [3, 4].

The IUA rate after miscarriages has been reported to be 6–20% [5–9], and the probability of developing IUAs after induced abortion ranges from 15 to 50% [6, 7, 10]. Previous studies have reported IUA rates; however, few have reported the impact of IUA on reproductive outcomes [6]. Dilatation and curettage (D&C) is a known risk factor for the development of IUA [5, 6], especially with profound negative suction pressure, or sharp, blind, or multiple curettages [5]. Therefore, increased awareness and recognition of IUA are crucial for early diagnosis and avoiding fibrotic scar formation [11]. The filmy adhesion bands in the early stage can be broken up by gently manipulating the hysteroscope with a distension pressure of 50–100 mmHg [4, 12]. However, once late adhesions form, harsh surgical steps are required for adhesiolysis, which are often accompanied by the risk of uterine perforation and further re-adhesion.

In a study [4] following hysteroscopic myomectomy for 1–3 months, IUA prevalence was 78% in the group without early lysis, and 0% in the early lysis group. The IUA rate was also related to myoma location and number. In addition, they [4] demonstrated that office hysteroscopy within 2 weeks of transcervical resection (TCR) is an efficient procedure for separating newly formed IUA. Previous studies [5, 7, 8] have advocated that post-abortion hysteroscopy is valuable for diagnosing acquired and congenital intrauterine pathologies, especially for women experiencing recurrent abortions.

No randomized control trials (RCTs) have analyzed the early hysteroscopic effect in preventing IUA, or fertility outcomes after IUA. We hypothesized that early second-look office hysteroscopy following induced abortion (suction D&C) with appropriate normal saline flushing might detect lesions earlier and further prevent IUA. The follicular phase of the first menstrual period after miscarriage was selected as a suitable time for early diagnosis of adhesions. In the present study, women with a further desire for conception were allocated to either early (first follicular phase of the last menstrual period following D&C) or late (6 months following D&C) hysteroscopy, and the effects were evaluated.

## Methods

### Participants

This is a single-center, prospective, two-armed, randomized controlled trial. Women were eligible to participate if they were 20–45 years of age, had a first-trimester induced abortion procedure in the previous month, and desired to conceive further. The participants were recruited from a medical center. Women who have had medical abortions, a known or possible increased risk of active pelvic infection (lower abdominal pain with tuboovarian complex mass or increased vaginal discharges), a history of more than three suction D&Cs, previous intrauterine myomectomy, previously confirmed intrauterine adhesions, or poor comprehension of spoken Mandarin were not eligible and excluded from this trial. If the patients became pregnant after enrollment, they were withdrawn from the trial after recording their fertility outcomes. If the rate of IUA in both groups is very low or if any group is found to have an inferior pregnancy outcome, the study will be considered stopped.

### Trial registration 

ClinicalTrials.gov, ID: NCT04166500. Registered on 2019-11-10. https://clinicaltrials.gov/ct2/show/NCT04166500.

### Randomization and blinding

After obtaining all baseline characteristics and evaluations, patients were randomly assigned to either the early hysteroscopy examination group (group A: women who underwent office hysteroscopy at the first follicular phase of the last menstrual period, and 6 months after D&C) or the late group (group B: women who underwent office hysteroscopy at 6 months after D&C) in a 1:1 ratio, in permuted blocks of four. A commissioner of the statistics center was responsible for generating block sizes and random codes using a computer-generated random-allocation sequence, which was concealed from the recruiting study assistant [13]. Given the nature of the intervention, it was impossible to blind the investigators or participants to group allocation.

### Sample size calculation

According to the literature, the probability of developing intrauterine adhesions after induced abortion ranges from 15 to 50% [6, 7, 10]. Considering the more severe cases of IUA, assuming a probability of 20% for the control group without any intervention after induced abortion (*p*_0_) and an expected improvement of *δ* = 15% (effect size = 0.48, moderate effect size), with a type I error rate (*α*) of 0.05 and a statistical power (1-*β*) of 0.8, a total of 60 participants are required per group based on the Z-test. Considering a 20% dropout rate, each group needs to include 75 participants, resulting in 150 participants in total.

### Induced abortion procedure: suction dilatation and curettage

The indications for choosing surgical abortion are as follows: (i) Fear of incomplete abortion from medical abortion. (ii) The patient wants to have the gestational tissue collected for chromosome study. (iii) Fear of heavy bleeding or uncertain cramping pain at home. The included criteria of suction D&C in this study were missed abortions before 13 gestational weeks. The standard procedure has been previously reported [14]. There was no preoperative misoprostol used. The patients who underwent D&C were anesthetized and placed in the lithotomy position. During the procedure, transabdominal ultrasonography was guided through a distended bladder to monitor the uterine axis and avoid unexpected uterine perforations and drastic blind curettage. After adequate Hegar dilation, the placental forceps and suction curette were gently applied to extract the gestational tissues. Intraoperative oxytocin was infused for better uterine contraction. A clear endometrial line and cavity were observed on ultrasound monitoring. Postoperative antibiotic prophylaxis was used.

### Hysteroscopy examination procedure

As per the study design, women allocated to the early group received two hysteroscopies (one at the first follicular phase of the menstrual cycle and the other at 6 months following D&C), and those in the late group received one (6 months following D&C). Hysteroscopy at 6 months was deferred in patients who got pregnant during the study or withdrew from the trial. All hysteroscopies were performed by the first and corresponding authors in the early proliferative phase using a Hysterovideoscope HYF (Olympus Optical Co., Tokyo, Japan). Office hysteroscopy was conducted using a flexible hysteroscope with a 3.8 mm diameter without mechanical or drug-induced cervical dilatation [15]. Neither anesthesia nor tenaculum usage was required. After sterilization, the hysteroscope was introduced, and saline solution (0.9% NaCl) with a distending media flow of 50–100 mmHg [16] was delivered into the uterine cavity from 60 cm above the patient. If a newly formed IUA was found, the doctor used the tip of the hysteroscope and the water flow force to separate the adhesion [4].

### Outcomes

The primary outcome was the IUA rate assessed using office hysteroscopy 6 months after D&C. IUA was observed and classified according to the American Fertility Society classification, with a mildest score of 1 to the most severe score of 12 [17].

Secondary outcomes included IUA score, menstrual amount (using pictorial blood loss assessment charts [PACs]) [18, 19], self-reported by patients and questionnaires), and fertility outcomes (pregnancy rates, abortion rates, ongoing pregnancy rates, and live birth rates). Ongoing pregnancy was defined as a viable pregnancy beyond 12 weeks of gestation. Abortion was defined as a pregnancy that terminated spontaneously before 12 weeks of gestation. Live birth was defined as the delivery of a viable fetus beyond 23 weeks of gestation [20].

### Statistical analysis

Continuous data are presented as mean ± standard deviation (SD). Mann–Whitney U tests were used to compare continuous data. Categorical variables, reported as proportions, were compared using the chi-squared test or Fisher’s exact test, as appropriate. All tests of significance were two-tailed, with *p* < 0.05 defined as being statistically significant. All statistical analyses were performed using SPSS for Windows, version 18.

## Results

**Fig. 1:**
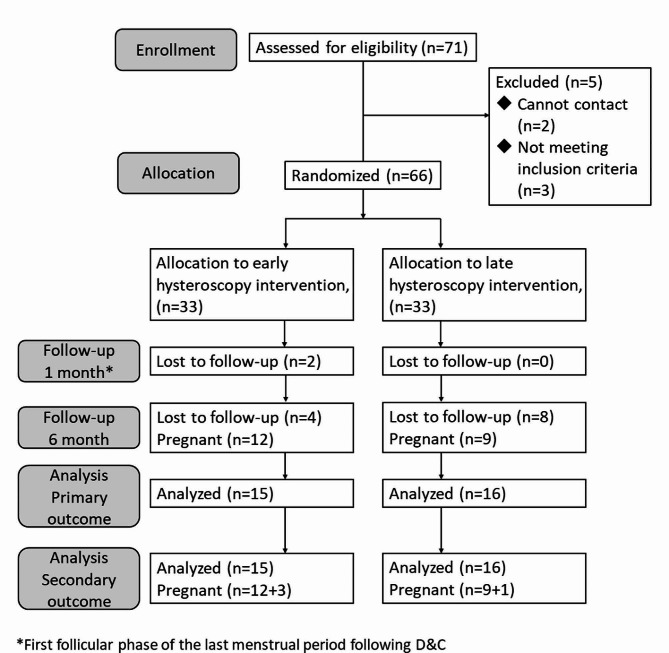
Flow chart showing the study’s recruitment, follow-up, and patient outcomes

A flowchart of the study is shown in Fig. 1. During the 3 years of study period, we enrolled 71 eligible participants from October 2019 to September 2022. Five women were excluded because they withdrew consent or were found to be ineligible after randomization. Sixty-six women were randomly allocated to receive either early (*n* = 33) or late hysteroscopy (*n* = 33). Two women in group A lost follow-up before 1st hysteroscopy exam. The follow-up rates were 81.8% (27/33) in the early intervention group and 75.8% (25/33) in the late group. The mean age was 37.6 ± 3.0 years in group A and 37.0 ± 4.8 years in group B (*p* = 0.57). Other baseline parameters, including BMI, and number of abortion histories, were comparable between the two groups (Table 1).

**Table 1:** Patient characteristics and clinical parameters

Variables	Early hysteroscopy (A) (*n* = 31)	Late hysteroscopy (B) (*n* = 33)	*P*-value
Female age, years	37.6 ± 3.0	37.0 ± 4.8	0.57
Body mass index, kg/m^2^	25.8 ± 2.0	28.1 ± 3.3	0.23
Number of para, *n*	0.3 ± 0.6	0.3 ± 0.5	0.92
Number of SA history, *n*	0.4 ± 0.7	0.3 ± 0.8	0.57
Number of AA history, *n*	1.2 ± 0.4	1.1 ± 0.3	0.53

Unless otherwise stated, all data are presented as mean ± SD or *n* (%)

Group A: early intervention group; Group B: late intervention group

SA, spontaneous abortion; AA, artificial abortion

No adverse events were observed during the trial period; no pelvic infections, uterine perforations, or significant vaginal bleeding were encountered.

### Primary outcome

In group A, 31 women underwent their first hysteroscopy, and 16.1% (5 of 31 cases) had mild-to-moderate IUA (extending < 1/3 uterine cavity; AFS scores were 2, 2, 2, 5, and 3). These five cases had all undergone induced abortion for the first time.

After 6 months of follow-up, 15 women in group A and 16 in group B were eligible for late hysteroscopic examination. The IUA rate was 0% in both groups (Table 2).

**Table 2:** Intrauterine adhesion rates in the two groups

Variables	Early hysteroscopy (A)	Late hysteroscopy (B)
IUA rate, *n*/*N* (%)		
Early hysteroscopy	5/31 (16%)	--
Late hysteroscopy	0/15 (0%)	0/16 (0%)

IUA, intrauterine adhesion. A: early intervention group; B: late intervention group

Data on the primary outcome were unavailable for 18 women in the early hysteroscopy group and 17 in the late hysteroscopy group, because some became pregnant, some were unwilling to return to the hospital for examination, and others were lost to follow-up.

### Secondary outcomes

The pregnancy rate was 55.6% (15/27) in the early intervention group and 40.0% (10/25) in the late intervention group (*p* = 0.26). The live birth, ongoing pregnancy, and abortion rates in group A were 60.0% (9/15), 60.0% (9/15), and 6.7% (1/15), respectively. The live birth, ongoing pregnancy, and abortion rates in group B were 30% (3/10), 50% (5/10), and 20% (2/10), respectively. Fertility and other secondary outcomes are summarized in Table 3. There were no significant differences between the two groups.

**Table 3:** Comparison of pregnancy and secondary outcomes between the two groups

Variables	Early hysteroscopy (A) (*n* = 27)	Late hysteroscopy (B) (*n* = 25)	*P*-value
Female age, years	37.6 ± 3.0	37.0 ± 4.8	0.57
Number of hysteroscopies, *n*	1.6 ± 0.6	0.6 ± 0.5	< 0.001*
Pregnancy, *n*/*N* (%)	15/27 (55.6)	10/25 (40.0)	0.26
Live birth, *n*/*N* (%)	9/15 (60.0)	3/10 (30.0)	
Ongoing pregnancy, *n*/*N* (%)	9/15 (60.0)	5/10 (50.0)	
Abortion, *n*/*N* (%)	1/15 (6.7)	2/10 (20.0)	
Menstrual amount, median points (IQR)	122 (81–280)	124 (89–169)	0.20
Menstrual duration, days	6.1 ± 1.6	5.5 ± 1.4	0.25

*P < 0.05; IQR, interquartile range. A: early intervention group; B: late intervention group

## Discussion

This novel prospective RCT design appears to provide the first documentation of the timing of office hysteroscopy in preventing IUA attributable to suction D&C. IUA detected early by hysteroscopy can disappear on late examination and become insignificant for future pregnancies. Regular early hysteroscopic examination following meticulous and sonography-guided D&C may not be necessary.

There are many protocols for non-surgical termination of pregnancy, like mifepristone and misoprostol [21, 22]. For the prevention of surgery and its complications, medical methods should be offered to the patient, and the final decision should be made with input from both sides. Blind and sharp curettages induce scar formation [5, 23]. Therefore, intraoperative transabdominal ultrasound monitoring is routinely performed at our hospital during suction D&C to ensure a clear endometrium and avoid unnecessary curettage [14]. In the present study, the IUAs formed were mild-to-moderate, extending to less than a third of the uterine cavity. This may explain why IUAs became insignificant at the late follow-up. According to Gilman et al., the estimated incidence of IUA following office hysteroscopy 2–4 months after a miscarriage was 6.3% [5]. The hysteroscopy evaluation period was similar to that of our early group, in which the IUA rate was 16.1%. Our study found that IUA detected early in hysteroscopy would disappear on the late examination and become insignificant for future pregnancies. Two women had mild IUA on early hysteroscopy and became naturally pregnant within 6 months. The other three women with IUA showed resolution on the second hysteroscopy. Endometrial repair and remodeling during the menstrual cycle [23] (breakdown bleeding and re-epithelialization) may facilitate lesion resorption.

**Fig. 2:**
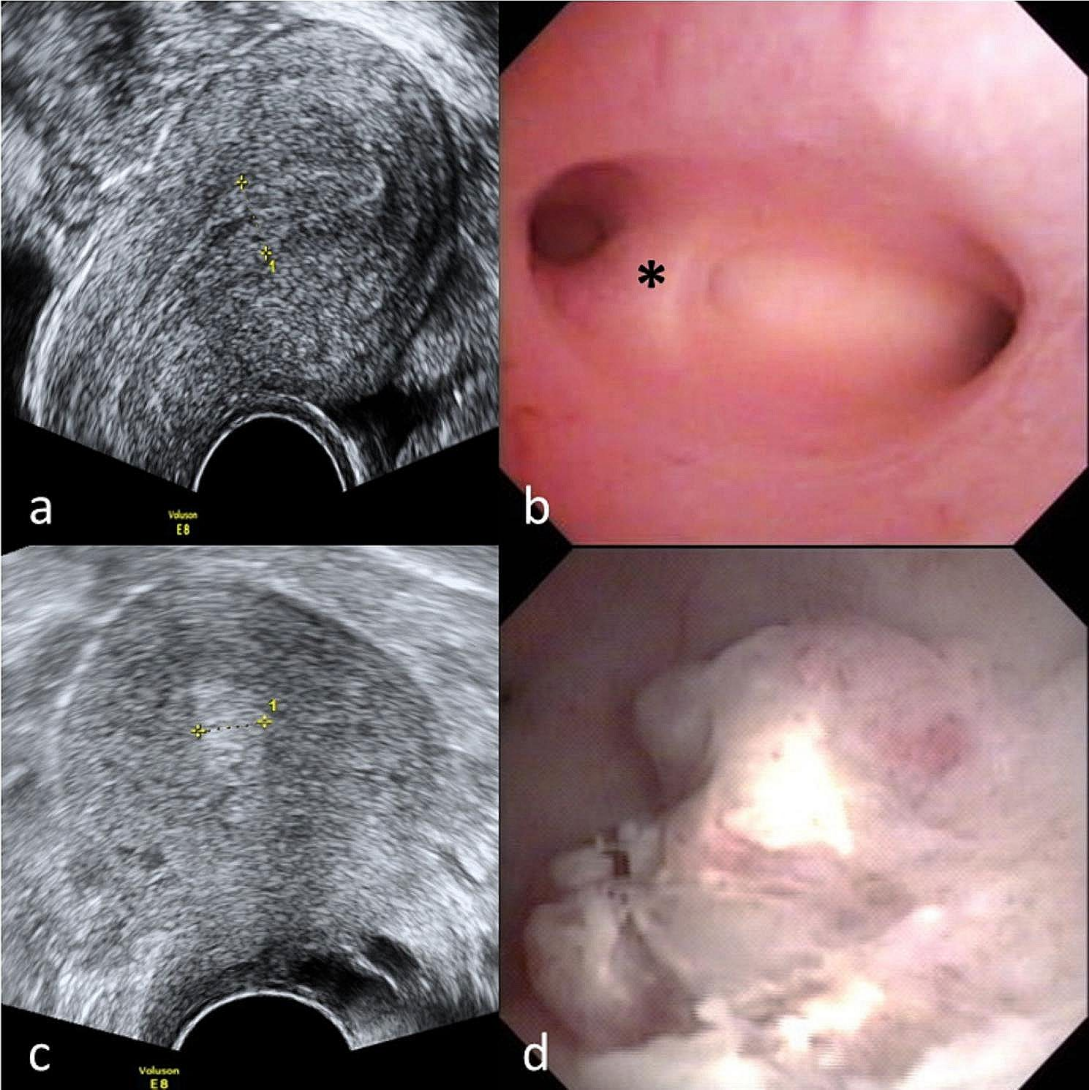
The transvaginal ultrasound and hysteroscopy finding with IUA (a&b) and RPOC (c&d). (**a**), A 34-year-old woman with G1P0AA1. The endometrial thickness was 0.79 cm. (**b**), The hysteroscopic image shows a dense IUA (asterisk) near the right ostium. The patient became pregnant during the follow-up period and did not receive a second hysteroscopy. (**c**), A 38-year-old woman with G1P0AA1. The endometrial thickness was 0.79 cm. (**d**), Hysteroscopy image revealing RPOC over the posterior cavity wall IUA, intrauterine adhesion; RPOC, retained products of conception

In this study, the signs and symptoms of IUA (*n* = 5) identified during the first hysteroscopy in group A were nonspecific. They did not manifest typical IUA clinical features [5], possibly because of mild uterine cavity involvement. The mean age was 35.6 years, there was a history of one abortion, the average menstrual blood was not decreased (the average points on Pictorial blood loss assessment charts was 178), and the menstrual duration was typically 7 days. The mean endometrial thickness was 0.88 cm with non-specific morphology. From this perspective, office hysteroscopy may be the examination of choice when IUA is suspected [24]. Figure 2 shows the transvaginal ultrasonography and hysteroscopy findings of IUA in group A.

Retained products of conception (RPOC) after pregnancy termination are not uncommon [25–27]. The presenting signs and symptoms include irregular vaginal bleeding and abnormal endometrial morphology on ultrasonography [25]. In the study, eight (8/31 = 25.8%) women were diagnosed with RPOC during early hysteroscopy, and six of them subsequently achieved pregnancy. In the late (6 months later) hysteroscopy, no women in group A and one in group B had RPOC. According to Raz N et al. [26], the see-and-treat approach during office hysteroscopy is as effective as operative hysteroscopy for RPOC smaller than 2 cm. Most RPOC after first-trimester abortion could be expectant management if there is no massive bleeding or hypervascularity [28]. The actual impact of RPOC following sonography-guided D&C is unclear. Whether early hysteroscopy to detect the lesion earlier is beneficial requires further investigation.

The pregnancy rate was higher in the early intervention group, although it was not significantly different from that in group B (Table 3). During early hysteroscopy, intrauterine lesions were found in 17 out of 31 cases, including retained products of conception (*n* = 8), intrauterine adhesions (*n* = 5), polyps (*n* = 1), submucosal myoma (*n* = 1), Mullerian duct anomaly (*n* = 1), and cesarean scar defect (*n* = 1). This would help us better understand the causes of miscarriage in patients, which would further facilitate subsequent pregnancies. Besides, during hysteroscopy, cervical dilation may correct stenosis, and uterine distention fluid flushes the uterine cavity while stimulating certain estrogen receptor factor expression [29, 30]. Whether early hysteroscopy enhances fertility requires further investigation.

The strengths of this study are its prospective design, homogeneous patient selection, and fertility outcome follow-up. Due to the extraordinary circumstances, patient recruitment and follow-up for this trial were significantly limited. First, we failed to recruit sufficient patients within the contract period, partially due to the coronavirus disease pandemic. Second, we were unable to retain patients due to some getting pregnant and cannot receive hysteroscopies. However, because very few IUAs were detected 6 months after suction D&C in this study, we decided to close the study, in compliance with the contract. Third, we excluded women with documented IUA, more than three abortions, and a history of TCR myomectomy. Patients who underwent spontaneous and/or medical abortions were also excluded. Fourth, hyaluronic gel or intrauterine balloon insertion may be an option for IUA prevention [31, 32]. However, we didn’t include these cohorts in this trial. Further studies may explore the role of hysteroscopy/ hyaluronic gel after multiple suction D&Cs and/or medical abortion. This preliminary report could be an impetus for others to attempt and verify it.

## Conclusions

This study suggests that early office hysteroscopy following well-monitored suction D&C with intraoperative sonography may not be necessary. Early hysteroscopy allowed early detection of intrauterine lesions. It is worth noting that the pregnancy outcomes had a favorable trend in the early hysteroscopy group but no statistically significant differences.

## Data Availability

All data generated or analyzed during this study are included in this article. Further inquiries can be directed to the corresponding author.

## Abbreviations

IUA: Intrauterine adhesions
D&C: Dilatation and curettage
TCR: Transcervical resection
PACs: Pictorial blood loss assessment charts
RPOC: Retained products of conception
